# Caregiver-assisted testing with HIV self-test kits for children 18 months and older: A GRADE systematic review

**DOI:** 10.1371/journal.pgph.0003588

**Published:** 2024-08-14

**Authors:** Kathleen McGee, Muhammad S. Jamil, Nandi Siegfried, Busisiwe Msimanga Radebe, Magdalena Barr-DiChiara, Rachel Baggaley, Cheryl Johnson

**Affiliations:** 1 Independent Public Health Consultant, Dakar, Senegal; 2 Regional Office to the Eastern-Mediterranean Region, World Health Organization, Cairo, Egypt; 3 Independent Clinical Epidemiologist, Cape Town, South Africa; 4 World Health Organization, Johannesburg, South Africa; 5 Global HIV, Hepatitis and STIs Programme, World Health Organization, Geneva, Switzerland; University of Toronto, CANADA

## Abstract

Caregiver-assisted testing using HIV self-test (CG-HIVST) kits has been proposed to enhance paediatric HIV case finding and contribute toward ending paediatric HIV/AIDS by 2030. We conducted a systematic review to assess the risks and benefits of CG-HIVST. We searched nine electronic databases and consulted experts to identify relevant articles through 5 February, 2022. Studies comparing CG-HIVST to other testing services among children over 18-months, or to no intervention, were included. Outcomes included uptake, acceptability, diagnostic accuracy, feasibility, HIV positivity, linkage to care, social harm, values and preferences, costs, and cost-effectiveness. Risk of bias was assessed using relevant Cochrane tools and certainty of evidence was evaluated with GRADE. Among 2203 screened articles, nine observational studies from sub-Saharan Africa were included. All studies used and assessed caregiver-assisted testing using oral fluid-based HIVST. In one non-randomized intervention study of 6062 children, overall CG-HIVST uptake was lower than other standard testing services (3.30% vs. 56.71%). In the same study, HIV positivity following CG-HIVST appeared lower or comparable to standard testing (RR = 0.44; 95% CI: 0.06, 3.20). Two single-arm studies reported high linkage to confirmatory testing (97.48%) and treatment initiation (97.7%) among children reported positive with CG-HIVST. Pooled positive predictive value was 36.72% across three non-randomized intervention studies. Reported social harms were rare, and acceptability appeared high among caregivers taking up the intervention, but feasibility was unclear as some reported anxiety in relation to reactive results. Evidence was appraised very low certainty. Average CG-HIVST costs varied widely and were consistently higher than standard testing services. CG-HIVST may be acceptable, but feasibility remains uncertain with potential higher costs. Current evidence favours standard testing for uptake and positivity. Low positive predictive values raise concerns about false positives and potential harm. Programmes should prioritize evidence-based approaches for paediatric case-finding, while research to fully evaluate this approach continues.

## Introduction

Reaching children with HIV to enable them to test and link to onward HIV prevention and care services is key strategy to end AIDS by 2030 [1]. There has been a substantial progress in addressing HIV among children through scale-up of prevention of mother-to-child transmission (PMTCT) services, infant diagnosis and treatment. However, only 57% of all children living with HIV in 2022 were accessing ART, resulting in approximately 660,000 children (0–14 years) living with HIV remain untreated [1]. Global progress is also far from achieving fewer than 15,000 new pediatric infections annually [2].

WHO recommends a package of evidence-based approaches for prevention and diagnosis of HIV infections in children [3]. These include improving access to PMTCT services for pregnant women, routinely offering HIV testing to pregnant women and prioritizing infant diagnosis for children born to parents who have not received PMTCT interventions. Subsequently, routinely offering testing to reach HIV exposed infants is critical for diagnosis and treatment [3]. Effective entry points for reaching infants and children who have been missed by infant diagnosis and routine follow-up testing include health facilities, including immunization, malnutrition, tuberculosis, and inpatient wards [4]. Family and household testing (also called provider-assisted referral or index testing) is also recommended for biological children of people with HIV. Virological testing is essential for infant diagnosis, however diagnosis of children above the age of 18 months should adhere to the standard national testing algorithms, often with use of three serology-based diagnostic tests [3].

HIV self-testing (HIVST) is an approach whereby an individual collects their own blood or oral-fluid specimen, performs the test and interprets their results. Since 2016, WHO has recommended HIVST to address gaps in HIV diagnosis [5]. Globally HIVST has been scaled up considerably as an effective approach to reach those who do not otherwise access services for testing. WHO guidance currently does not recommend caregiver-assisted testing with HIVST kits (CG-HIVST), defined as an HIV testing approach whereby a parent or guardian performs HIV testing on a child, generally as part of family-based index testing, using a quality-assured self-test. In recent years, some programs have explored the utility of CG-HIVST as an additional approach to identify undiagnosed children [6–14]. In 2022, we conducted a systematic review to assess the potential risks and benefits of CG-HIVST to inform the development of WHO guidelines and recommendations. This paper presents the work and findings that were presented and used to inform WHO’s guideline development group in November 2022.

## Materials and methods

The review protocol was registered online in the International prospective register of systematic reviews (PROSPERO Registration number: CRD42022302617). No deviations from the registered protocol were made.

### Search strategy and selection criteria

We searched nine electronic databases including Ovid Medline, Embase, CINAHL Plus, EconLit, Global Health, PsycInfo, Cochrane Library, Web of Science, and Scopus through 5 February 2022. We verified secondary references on previously published review articles relevant to HIVST and contacted experts in the field to identify additional articles and abstracts. As previously stated, this paper presents the results as they were presented to WHO’s guideline development group in November 2022. While the search results have not been updated since 2022, a recent search of the literature has revealed no new publications regarding CG-HIVST, as of December 2023. This additional search included a review of the databases mentioned earlier to verify that no new publications relevant to our study were available during this period.

The search strategy was adapted to each database using key terms “HIV”, “self-test” (S1 Table)). No restrictions were placed on location of the intervention or language of article. Two reviewers screened articles and differences in judgement were resolved by other review team members. To be included in the review, a study had to directly compare CG-HIVST to any other HIV testing services for children, or to no intervention. Studies offering caregivers the choice between CG-HIVST and other pediatric testing modalities were included. Eligible studies were limited to children 18 months of age up to 18 years of age, as children younger than 18 months should be diagnosed based on virological testing as per WHO recommendations [3]. Studies also had to report on one or more of the following outcomes: 1) HIV testing uptake (defined as proportion of children tested for HIV in study period among those enrolled. This outcome measures the actual uptake of CG-HIVST among the enrolled population of children. Note that one caregiver may have multiple children tested); 2) Acceptability of CG-HIVST (proportion of caregivers who accepted and chose the CG-HIVST testing approach. This outcome reflects the willingness of caregivers to opt for the CG-HIVST method); 3) Diagnostic accuracy (measured by sensitivity, specificity, positive predictive values (PPV), and negative predictive values); 4) Feasibility (proportion of caregivers who correctly administered CG-HIVST) and usability (proportion of caregivers who reported CG-HIVST easy to administer); 5) HIV positivity (proportion of children tested and confirmed positive); 6) linkage to care (proportion of children tested reactive linked to confirmatory testing, clinical assessment, or treatment); 7) social harm/adverse events (occurrence and reporting of any harm or undesirable experience occasioned by the intervention to children and/or caregivers); 8) values and preferences (reported facilitators and barriers associated with the intervention); 9) costs and cost-effectiveness.

### Data appraisal and synthesis

Data was extracted by one reviewer (KM) using a standardized data extraction form. Study authors were contacted when additional data or clarification was needed. Pair-wise meta-analysis was planned, however, due to methodological heterogeneity across studies, this was not possible and quantitative and qualitative data was summarized descriptively. When values and preferences or resource use were reported by included studies they were descriptively summarized and reported. Studies reporting on costs or cost-effectiveness were also adjusted to and reported in 2021 United States Dollars. Risk of bias (RoB) was assessed using the Risk of Bias in Non-randomized Studies–of Interventions (ROBINS-I) tool [15]. Studies reporting on usability and diagnostic accuracy were also assessed using the Quality Assessment of Diagnostic Accuracy Studies (QUADAS-2) tool [16]. In accordance with WHO guideline development practice, we report the results using Grading of Recommendations, Assessment, Development and Evaluations (GRADE) recommended language which integrates the clinical importance of the estimate of effect with the overall certainty of the estimate [17]. Following guidance from GRADE Handbook, overall certainty of evidence was rated by appraising risk of bias, level of imprecision, indirectness, inconsistency and other considerations [18]. Full RoB and GRADE Quality Assessment is available in (S1 Text and S2 Table).

### Role of the funding source

The funder of the study had no role in study design, data collection, data analysis, data interpretation, or writing of the report.

## Results

**Fig 1:**
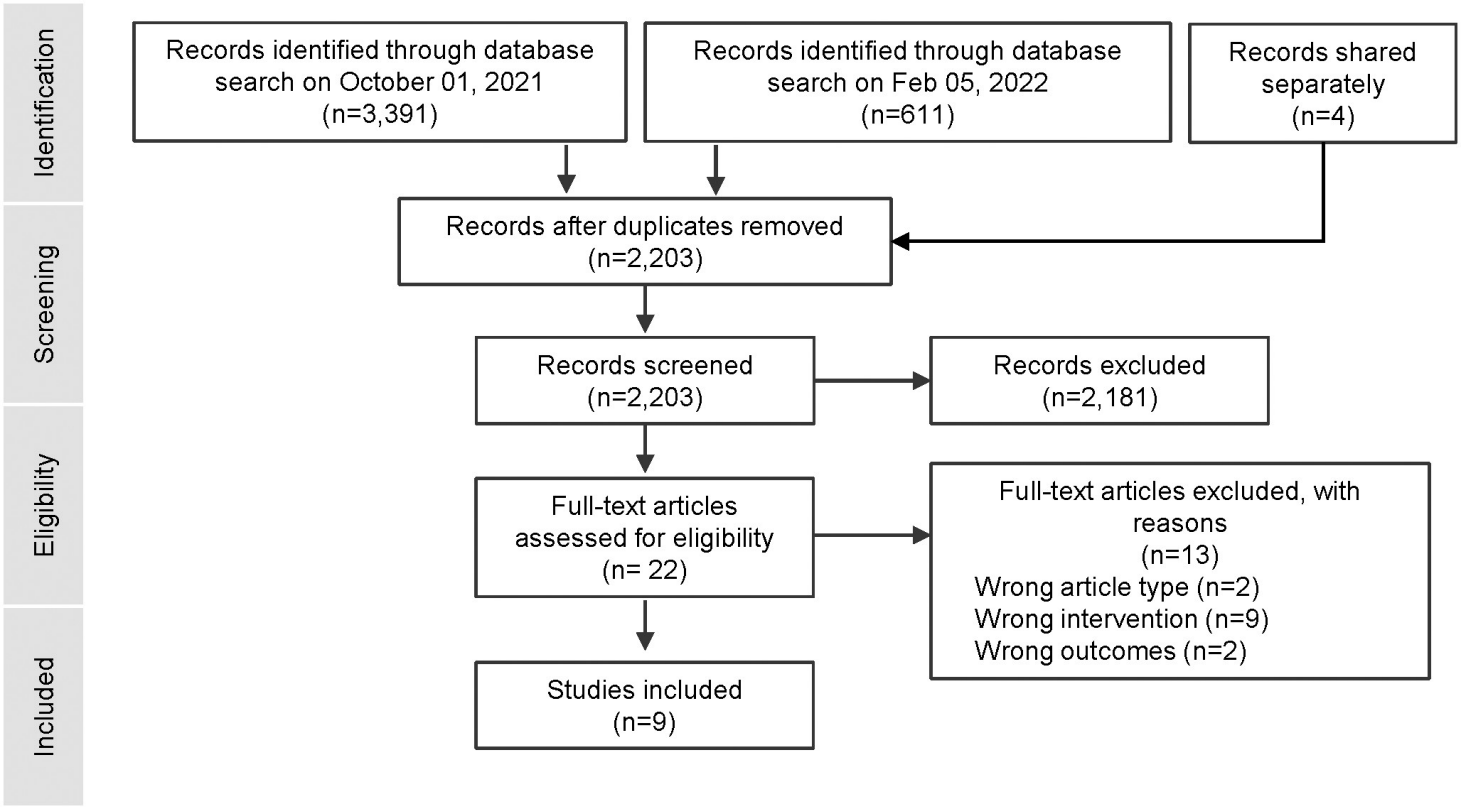


Searches were conducted on October 1st, 2021, and repeated on February 5th, 2022. Update search conducted in December 2023 revealed no new publications. Four records were identified through field experts. After duplicates were removed, the combined searches yielded 2,203 citations, of which, nine observational studies were included in the review (see “Fig 1”).

Across all studies there were 6,916 caregivers with HIV and 13,840 children of unknown HIV status. All studies used oral fluid-based HIVST and were among children of caregivers with HIV (aged 18 months to 18 years). All Studies were in sub-Saharan Africa (Zimbabwe, Kenya, Uganda, and Zambia); with all but one related to two intervention trials: Bridging the Gap in HIV testing and care for children (or B-GAP) [6], which was conducted in Zimbabwe, and the Faith-based Action for Scaling up Testing and Treatment for Epidemic Response (or FASTER), which was conducted in Uganda and Zambia (see Table 1) [10, 11].

**Table 1:** Summary of included study characteristics.

Study ID	Study Name	Country	Population	Reported Outcomes	Arms
Chikwari et al, 2021 [6]	B-GAP	Zimbabwe	2870 index caregivers & 6062 children 2–18 yrs	Uptake, Positivity, Linkage to care, Acceptability, Social harm	1. Facility-based HTS
					2. Home-based HTS
					3. CG-HIVST
Vasantharoopan et al, 2021 [7]			2870 index caregivers & 6062 children 2–18 yrs	Costs	1. Facility-based HTS
					2. Home-based HTS
					3. CG-HIVST
Chikwari et al, 2021 (b) [8]			400 index caregivers & 786 children 2–18 yrs	Feasibility, Diagnostic accuracy	1. CG-HIVST with demonstration
					2. CG-HIVST with no demonstration
Rainer et al, 2021 [9]			20 index caregivers & 11 adolescents under 18 yrs	Values and Preferences	1. CG-HIVST
Tumwesigye et al, 2022 [10, 11]	FASTER	Uganda	2393 index caregivers & 4964 children 18mo-14 yrs	Uptake, Positivity, Linkage to care, Acceptability, Feasibility, Accuracy	1. CG-HIVST
Stecker et al, 2022 [10]		Zambia	1656 index caregivers & 2814 children 18mo-14 yrs	Uptake, Positivity, Linkage to care, Acceptability, Feasibility, Accuracy	1. CG-HIVST
Kagaayi et al, 2022 [12]		Uganda & Zambia	4049 index caregivers & 7778 children 18 months-14 yrs	Costs	1. CG-HIVST
Gross et al, 2022 [13]				Social Harm	
Neary et al, 2022 [14]	STEP-UP	Kenya	32 index caregivers & 38 health care workers	Values and Preferences	1. CG-HIVST

B-GAP: Bridging the Gap in HIV testing and care for children; FASTER: Faith-based Action for Scaling up Testing and Treatment for Epidemic Response; STEP-UP: Saliva Testing and Video Information to Expand Uptake of Paediatric HIV Testing; CG-HIVST: Caregiver-assisted testing with HIVST kits; HTS: HIV testing services

B-GAP, a non-randomized intervention study without a control group, allowed caregivers to choose among three index-testing approaches for their children: 1) standard facility-based testing, 2) home-based testing by a lay provider, and 3) CG-HIVST. Chikwari 2021 presented the main study findings, including uptake, positivity, linkage to care, acceptability and social harm. B-GAP also includes a cost analysis [7], a study on feasibility and diagnostic accuracy [8], and a qualitative study on values and preferences [9]. The second study, FASTER, includes two parent trials, Tumwesigye 2022 [10, 11] and Stecker 2022 [10], both single-arm intervention studies, along with a cost analysis [12], and an additional analysis on the effects of screening for social harm [13]. Finally, Neary 2022 [14], conducted a qualitative study as part of the larger STEP-UP studies [19], and used focus group discussions to explore the values and preferences of caregivers and health care workers.

### HIV testing uptake

A single non-randomized intervention study [6] directly compared and reported HIV testing uptake between CG-HIVST and provider-assisted index testing. In the study, caregivers were offered the choice among three testing options for children: CG-HIVST, home-based testing by lay provider and standard facility-based testing by health worker. Out of 6,062 eligible children enrolled in the study, 3.3% (n = 197/6062) were tested by CG-HIVST. In comparison, 31.61% (n = 1916/6062) tested in a facility by a health provider, and 25.11% (n = 1522/6062) tested at home by a lay health-worker. Certainty of this evidence for uptake was very low due to an extremely serious risk of bias.

In the same study, when excluding children whose caregivers refused any testing option (non-ITT approach), uptake was highest for children whose caregivers opted for home-based HIV testing at 69% (n = 1026/1487), followed by 65.67% of children whose caregivers selected CG-HIVST (n = 197/300), and by 52.13% of children selected into facility-based HIV testing (n = 1845/3539). While denominators reflect the number of children selected for testing option, there were instances of crossovers between study arms. Certainty of this evidence was very low due to an extremely serious risk of bias. Potential in-study heterogeneity related to child sex and age was explored using subgroup analysis, but no differences in uptake was found (additional analysis available in (S3 Table).

### Acceptability of HIV testing

Acceptability of CG-HIVST was reported in two single-arm intervention trials [10]. Overall, both studies reported high acceptability among caregivers who opted into CG-HIVST (96.91%, 3924/4049) and among caregivers who performed the self-test on their children (94.02%, 3807/4049). Evidence was graded as uncertain because study enrolment procedures introduced risk of bias. Prior to study enrolment, study teams approached index caregivers and offered them a choice of HIV testing services for their children. Caregivers who opted for CG-HIVST were enrolled and remaining caregivers were re-directed to alternative testing modalities. *N*either study was able to provide information on the proportions of approached caregivers who accepted or declined CG-HIVST.

### Diagnostic accuracy

Diagnostic accuracy was reported in three studies [8, 10, 11] *(see* Table 2*)*, comparing caregivers’ interpreted results of CG-HIVST to the results of blood-based rapid HIV testing performed by researchers. In one study [8], specificity of CG-HIVST was high (98.6%; 95%CI: 97–99%) but sensitivity, while high, had a very large confidence interval (100%, 95%CI: 40–100%) due to a small sample size and very few children testing positive. Negative predictive value was 100%. Two other studies [10, 11] reported performance and accuracy measures, including positive predictive values (PPV) and the number of discrepant results. However, true sensitivity and specificity of CG-HIVST could not be estimated as only children testing positive received confirmatory testing. Using total true positives and total false positive results, the pooled positive predictive value (PPV) of all three studies [8, 10, 11] was 36.72% (range: 32.7–61.1%); indicating there could be a substantial risk of false positive results. Due to the absence of reported true negatives in two studies [10, 11], it was not possible to calculate a weighted PPV average across studies. All studies were treated equally to avoid potential bias.

**Table 2:** Summary of evidence on accuracy and performance of caregiver-assisted testing with HIVST kits.

	Chikwari, 2021 (b) * [8]			Tumwesigye, 2022^±^ [11]		Stecker, 2022 **^±^ [10]		
	Provider Reactive	Provider Negative	Provider Inconclusive	HTS positive	HTS negative	HTS positive	HTS negative	LTFU
CG-HIVST Reactive	4	8	1	32	66	11	7	3
CG-HIVST Negative	0	567	0	-	-	-	-	-
CG-HIVST Inconclusive	0	1	6	-	-	0	2	8
Specificity	98.61% (95% CI: 97%-99%)							
Sensitivity	100% (95% CI: 40%-100%)							
PPV	33.33%			32.65%		61.11%		
NPV	100%							

* Results different than those published. Results reported here were confirmed and validated by study authors;

** Includes additional data from an unpublished report;

^±^ Screened negatives were not followed for confirmatory testing.

HTS: HIV testing services; LTFU: Loss-to follow-up; CG-HIVST: caregiver-assisted HIVST; PPV: positive predictive value; NPV: negative predictive value; CI: confidence interval

Additional analysis from one study in Uganda [11] reported that implementing a refresher training course to staff did reduce the number of discrepant results over time. In this study, it was reported positive predictive values increased from 23.33% in weeks 1–8 to 47.37% in weeks 9–25.

### Feasibility and usability

Feasibility of CG-HIVST was reported in three trials [8, 10]. In one study [8], the majority caregivers who received an in-person demonstration on how to test children with an HIVST kit were observed to correctly administer the test (92.4%, 145/157). However, in this same study, caregivers who did not receive a demonstration appeared to have more difficulty with only 78.0% (490/629) able to correctly administer the test. In two studies [10], most caregivers receiving demonstrations felt testing was easy to administer (96.95%, 2636/2719) but three-quarters 75.30% of caregivers who did not receive demonstration felt testing was easy to administer. The certainty of evidence was very low.

### HIV positivity

HIV positivity was reported by one study with a comparative design [6]. There was very low certainty that HIV positivity was reduced in the CG-HIVST group compared to other index-testing options, but this estimate ranged from a reduction in HIV positivity of 94% compared to the other index-testing approaches to a threefold increase in HIV positivity (RR = 0.44; 95% CI:0.06, 3.20). In two additional single-arm studies, the pooled HIV positivity was 0.57% (n = 43/7608) [10].

### Linkage to further confirmatory testing, clinical assessment and treatment

Linkage to further testing and care following CG-HIVST was reported in two single-arm trials [10]. In one study in Uganda, all children with a reactive result (n = 98) received further testing within 1-month of follow-up. However, in the other study in Zambia, 86% of children with a reactive result (n = 21) received further testing within 1-month of follow-up. Across both studies, among children receiving confirmatory testing and diagnosed with HIV, linkage to care was 97.5% (116/119).

### Potential social harms

Potential harm related to CG-HIVST was reported in three studies [6, 10, 13] In one study [10], there were reports of minor adverse events related to CG-HIVST, such as children experiencing gum pain (n = 6/9), bleeding gums (n = 3/9), or gum itching (n = 1/9).

There were no reports of physical harm or abuse to caregivers or children in any of the studies. In two single-arm studies [13], caregivers were pre-screened for factors associated with violence or harm, rendering the results uncertain.

### Values and preferences

Across four studies, caregiver-assisted testing with HIVST kits was well-liked by caregivers among those who opted into the approach[9, 10, 14], with many caregivers feeling it was private, convenient, and could protect from potential stigma and discrimination. Some caregivers expressed concerns about their ability to correctly administer HIVST and about the accuracy of the approach [9, 10, 14], as well as their ability to deliver post-test messages to children and to handle a reactive result [9, 14]. For this reason, many caregivers desired the presence of a provider or counsellor when testing children [9, 10, 14]. Some caregivers also felt that greater education and sensitization about CG-HIVST was needed, along with efforts to optimize the intervention by providing better demonstrations on how to perform the test and to improve supply and access to HIVST kits [10].

### Resource use

Two studies reported on resource use [7, 12] and are summarized in (S4 Table). Overall, the average cost per child tested ranged from $5.12 in Zambia to $154.23 in Zimbabwe, and the average cost per child diagnosed range from $869.00 in Uganda to $2,468.68 in Zimbabwe.

One study compared costs across different testing modalities for children and adolescents [7]. In this study, the average cost per child tested using standard testing approaches ranged from $14.58 to $17.67, whereas the incremental average cost of CG-HIVST to standard testing range from $43.48 to $154.23. When this was compared to other standard index-testing approaches, the incremental cost per child tested of CG-HIVST was three to eleven times greater than standard testing approaches for children and adolescents.

The key cost drivers across studies were HIVST commodities, as well as personnel used to deliver support and demonstrations. In one study, personnel costs ranged between 39.7% and 50.3% and supervision costs ranged between 25.7% and 29.1% of total costs [7]. Two other studies had lower personnel costs (15–16% of total), but reported higher cost HIVST kits which took up between 41% and 51% of the total costs [12].

Average costs per child tested and per child diagnosed were sensitive to testing uptake and positivity. Because HIV positivity was low across all studies, this contributed to high average unit costs per child diagnosed.

### Risk of bias

In the risk of bias assessment (summarised in Figure A, Tables A and B in S1 Text), several critical and serious risks of bias were identified that affected the certainty of evidence. Overall, measures of testing uptake, positivity, acceptability, feasibility and social harm were contingent on caregivers’ self-reporting. Notably, all three studies exhibited a significant gender imbalance among caregiver participants, with a higher proportion of female caregivers opting for CG-HIVST (79% female in Chikwari 2021, 73% female in Tumwesigye 2022, 88% female in Stecker 2022). Multivariate analyses further confirmed women were more likely to uptake, potentially rendering testing uptake and ensuing outcomes appear greater among these study populations. The ROBINS-I tool also highlighted risks of bias in the selection of participants, such as in Tumwesigye 2022 and Stecker 2022 [10] where caregivers opting for other testing options were directed to other services and excluded from the study. Chikwari 2021 [6] was found to have critical bias due to deviations from caregivers’ originally selected testing modalities and an overall attrition rate of 21%.

## Discussion

This systematic review set out to assess the risks and benefits of CG-HIVST and explore whether CG-HIVST may be an additional, safe and effective approach to increase diagnosis of children living with HIV. While revealing challenges in uptake, diagnostic accuracy, and feasibility, the review also demonstrated high acceptability and a low risk of harm. However, the review found that available evidence remains at very low certainty, limiting the ability to make robust conclusions.

The available evidence, albeit limited to a single comparative study [6] suggests that uptake of CG-HIVST among caregivers was comparatively low, with only 3.3% of eligible children tested using this approach. Other standard index-testing approaches showed higher uptake rates, including facility-based testing by health providers and home-based testing by lay health workers. While the single-arm studies from Uganda and Zambia [10] signaled CG-HIVST may be highly acceptable, they did not report on how many caregivers approached to join the studies declined CG-HIVST, suggesting possible preference for alternate options. While HIV positivity appeared to be low across studies (less than 1%), particularly in the studies’ high HIV burden settings and among children of parents living with HIV, the effects of CG-HIVST on positivity remain uncertain.

No cases of physical harm or abuse to children or caregivers were reported in all three intervention studies [6, 10] however evidence was limited as two of the three studies [10] excluded participants at-risk of social harm prior to study enrolment [13]. Furthermore, low positive predictive values raise concerns that potential false-positive results may increase the risk of harm, users’ costs when seeking confirmatory testing, and erode the confidence of users in CG-HIVST. These same concerns are echoed in qualitative interviews, where caregivers reported concerns on accuracy, how to handle reactive results, and expressed desire for post-test provider or counsellor support. It is important to note that studies directly comparing the diagnostic accuracy of blood-based and oral-fluid HIV self-testing (OF-HIVST) among adult populations have found OF-HIVST to have very high sensitivity and specificity (Figueroa et al 2018), with recent studies reporting PPVs of 95.24% [20] and 98.13% [21]. The tests used in these studies were also prequalified and reported 99.4% sensitivity and 99.0% specificity [22]. This suggests that the lower accuracy observed in this review may be due to challenges in implementing the approach among children and/or caregivers, especially in settings with very low HIV prevalence. While these studies focused on a population of biological children of people with HIV, HIV positivity across the three studies stood at less than 1%, directly affecting the PPV and thus further indicating the need for HIV testing strategies to be appropriately designed and targeted for paediatric populations.

Feasibility appeared to be high when caregivers received demonstrations on how to correctly administer HIV self-testing to their children, but this adds to costs (including test costs) and staff time. While such approaches have been valuable in the roll-out of existing HIVST approaches among adult populations and should not be discounted, the cost of this approach as part of CG-HIVST may be a limiting factor. In Zimbabwe, CG-HIVST incurred incremental costs three to eleven times higher than standard testing, driven largely by the low uptake and low positivity of the approach. Implementing CG-HIVST at scale may pose challenges, especially in resource-constrained settings. While CG-HIVST may increase access to some, the intervention’s high costs and low yields may lead to de-prioritization or fewer resources for other more effective testing modalities. Until further evidence demonstrate CG-HIVST to be safe and effective, programs may need to prioritize recommended pediatric testing approaches, leveraging existing and effective strategies for reaching children such as PMTCT, malnutrition, TB, and immunization services, which, to date have not been fully maximized [23]. Given the substantial gaps among children, the suboptimal implementation of these evidence-based testing approaches to reach children and adolescents needs to be addressed urgently through updated national strategies in sub-Saharan Africa where, in the most recent population-level study, close to 40% of children had undiagnosed HIV [24].

This review was significantly limited by the number of studies that directly compared CG-HIVST to other standard pediatric index-testing services. Meta-analysis was not feasible, and the overall certainty of evidence was very low due to very high risks of bias as well as the indirectness of the evidence, caused by study enrolment criteria and procedures. Future systematic reviews and meta-analyses will need studies with comparative study designs to better understand the relative risks, benefits, and preferences between CG-HIVST and other pediatric testing modalities. While randomized controlled trials are considered the gold standard, cohort, pre- and post- and other observational study designs using rigorous methods to minimize bias due to selection of participants and attrition would be valuable. The areas of greatest uncertainty that require further attention include the accuracy, resource use, and the actual impact of CG-HIVST on uptake of HIV testing, case-finding and treatment in the undiagnosed paediatric population compared to existing strategies. Future studies may also want to explore considerations between different age groups including potential issues on coercion, age of consent, provision of first line support services to children and families where abuse or harm are identified, and implementation of the approach for adolescents and older children. For adolescents, provider or peer-delivered approaches [25–27] may be preferable, in settings where age of consent polices allow HIVST distribution to adolescents. It would also be valuable for studies to better characterize and understand differences particularly among populations that decline the intervention. This requires study designs that compare outcomes and insights from different population groups, including those who find the testing approach acceptable and those who decline it. Resource use is increasingly important with policy shifts towards sustainability and decreased funding from donors. The cost per diagnosis was high in the studies included in this review and it is important to identify opportunities to optimize limited resources for paediatric case-finding.

Based on the findings of this review, the guidelines development group convened by WHO assessed the evidence not sufficient and conclusive at this time to make a recommendation for or against CG-HIVST. Since the systematic review was conducted and presented, no additional studies on CG-HIVST have been published, as far as authors are aware. Lack of evidence emphasizes the need for ongoing research. While the safety and effectiveness of CG-HIVST remains very uncertain and needs to be further investigated, greater efforts are needed to better reach children and adolescents living with HIV and to link them to care. Investment and scale-up of effective pediatric and family-centered testing approaches remain crucial and must be delivered beyond traditional service delivery points. It is important that the global HIV response prioritizes existing pediatric case-finding interventions until further evidence is available.

## Supporting information

S1 Table Full search term for Ovid Medline. This appendix contains the detailed search strategy used for identifying relevant studies in the Ovid Medline database. It includes specific search terms and the combinations used.
(DOCX)

S2 Table GRADE assessment. This appendix contains the GRADE evidence profiles for each outcome assessed in the systematic review, detailing the certainty of evidence and summary of findings.
(DOCX)

S3 Table Within-study subgroup analyses. This appendix contains the subgroup analyses for each outcome assessed by gender and children age groups.
(DOCX)

S4 Table Summary of costs. This appendix contains the summary of the reported unit costs for caregiver-assisted HIV self-testing, expressed in 2021 USD. Comparative unit costs for alternative index-testing modalities were included when reported.
(DOCX)

S1 Text Risk of bias assessment. This appendix contains the risk of bias assessment for each included study using the ROBINS-1 and QUADAS-2 assessment tools. It includes summary results as well as a detailed justification of the assessment for each study and each domain.
(DOCX)

S1 Checklist This appendix provides the PRISMA checklist, ensuring compliance with the systematic review reporting standards. (DOCX)
