# Supplementary material for: Caregiver-assisted testing with HIV self-test kits for children 18 months and older: A GRADE systematic review
Source: PLOS Glob Public Health. 2024 Aug 14;4(8):e0003588. doi: 10.1371/journal.pgph.0003588 (PMC11324119; doi:10.1371/journal.pgph.0003588)
Supplement: S1 Table — This appendix contains the detailed search strategy used for identifying relevant studies in the Ovid Medline database. It includes specific search terms and the combinations used. (DOCX) [file pgph.0003588.s001.docx]

### **S1 Table. Full search term for Ovid Medline**

| Ovid Medline | |
| --- | --- |
| Action | **Term** |
| 1 | exp HIV Infections/ |
| 2 | exp HIV/ |
| 3 | hiv.ti,ab |
| 4 | "hiv1".ti,ab. |
| 5 | "hiv2".ti,ab |
| 6 | “hiv type 1".ti,ab. |
| 7 | "hiv type 2".ti,ab. |
| 8 | human immunodeficiency virus.ti,ab |
| 9 | human immunedeficiency virus.ti,ab. |
| 10 | human immuno-deficiency virus.ti,ab |
| 11 | human immune-deficiency virus.ti,ab |
| 12 | (human immun* adj3 deficiency virus).ti,ab |
| 13 | acquired immunodeficiency syndrome.ti,ab |
| 14 | acquired immunedeficiency syndrome.ti,ab |
| 15 | acquired immuno-deficiency syndrome.ti,ab |
| 16 | acquired immune-deficiency syndrome.ti,ab |
| 17 | (acquired immun* adj3 deficiency syndrome).ti,ab |
| 18 | Sexually Transmitted Diseases, Viral/ |
| 19 | or/1-18 |
| 20 | (sample adj1 collect*).ti,ab |
| 21 | Dried Blood Spot Testing/ and (home* or remote or personal or self*).ti,ab |
| 22 | (dried blood spot adj3 (home* or remote or personal or self*)).ti,ab |
| 23 | (alternative adj3 test*).ti,ab |
| 24 | (option* adj1 test*).ti,ab |
| 25 | ((Home* or self* or mail*) adj3 (collect* or sampl* or specimen* or test* or kit)).ti,ab |
| 26 | or/20-25 |
| 27 | 19 and 26 |
