# Supplementary material for: Caregiver-assisted testing with HIV self-test kits for children 18 months and older: A GRADE systematic review
Source: PLOS Glob Public Health. 2024 Aug 14;4(8):e0003588. doi: 10.1371/journal.pgph.0003588 (PMC11324119; doi:10.1371/journal.pgph.0003588)
Supplement: S2 Table — This appendix contains the GRADE evidence profiles for each outcome assessed in the systematic review, detailing the certainty of evidence and summary of findings. (DOCX) [file pgph.0003588.s002.docx]

### **S3 Table. GRADE Assessment**

| **Certainty assessment** | | | | | | | **№ of patients** | | **Effect** | | **Certainty** | **Importance** |
| --- | --- | --- | --- | --- | --- | --- | --- | --- | --- | --- | --- | --- |
| **№ of studies** | **Study design** | **Risk of bias** | **Inconsistency** | **Indirectness** | **Imprecision** | **Other considerations** | **Caregiver-assisted HIV self-tests** | **Standard of Care** | **Relative (95% CI)** | **Absolute (95% CI)** |  |  |
| **Testing Uptake (among children enrolled)** | | | | | | | | | | | | |
| 1^1^ | observational studies | extremely serious^a^ | not serious | not serious | not serious | none | One cohort study with comparator demonstrated that, among all children enrolled in the study, uptake of caregiver-assisted HIV testing with HIVST kit was 3.30% (n=200/6062), while uptake of provider-administered HIV testing was 56.71% (n=3,438/6062).  Caregivers had an option of 3 testing methods. Testing uptake was highest for facility-based testing at 31.61% (n=1916/6062), followed by home-based testing at 25.11% (n=1522/6062), followed by caregiver-assisted HIV testing with HIVST kit at 3.30% (n=200/6062). ^b^ | | | | ⨁◯◯◯ Very low | IMPORTANT |
| **Testing uptake (Caregiver-assisted HIVST vs. Provider-administered HTS, among selected testing regimen)** | | | | | | | | | | | | |
| 1^1^ | observational studies | extremely serious^c^ | not serious | serious^d^ | not serious | none | 197/300 (65.7%) ^e^ | 2871/5026 (57.1%) ^e^ | **RR 1.15** (1.06 to 1.25) | **86 more per 1,000** (from 34 more to 143 more) | ⨁◯◯◯ Very low | IMPORTANT |
| **Testing uptake (Caregiver-assisted testing with HIVST vs. Facility-based HTS, among selected testing regimen)** | | | | | | | | | | | | |
| 1^1^ | observational studies | extremely serious^c^ | not serious | serious^d^ | not serious | none | 197/300 (65.7%) ^e^ | 1845/3539 (52.1%) ^e^ | **RR 1.26** (1.15 to 1.38) | **136 more per 1,000** (from 78 more to 198 more) | ⨁◯◯◯ Very low | IMPORTANT |
| **Testing uptake (Caregiver-assisted testing with HIVST vs. Home-based HTS, among selected testing regimen)** | | | | | | | | | | | | |
| 1^1^ | observational studies | extremely serious^c^ | not serious | serious^d^ | serious^f^ | none | 197/300 (65.7%) ^e^ | 1026/1487 (69.0%) ^e^ | **RR 0.95** (0.87 to 1.04) | **34 fewer per 1,000** (from 90 fewer to 28 more) | ⨁◯◯◯ Very low | IMPORTANT |
| **Acceptability (1): Caregiver accepted intervention** | | | | | | | | | | | | |
| 2^2,3^ | observational studies | extremely serious^g^ | not serious | serious^h^ | not serious | none | Two cohort single-arm studies where eligible index-caregivers accepted caregiver-assisted HIV self-testing for their children at a pooled rate of 96.91% (n=3924/4049) | | | | ⨁◯◯◯ Very low | CRITICAL |
| **Acceptability (2): Caregiver tested children with HIVST** | | | | | | | | | | | | |
| 2^2,3^ | observational studies | extremely serious^g^ | not serious | serious^h^ | not serious | none | Two cohort single-arm studies where eligible index-caregivers tested their chidren with an HIV self-test at a pooled rate of 94.02% (n=3807/4049) | | | | ⨁◯◯◯ Very low | CRITICAL |
| **Accuracy (Specificity)** | | | | | | | | | | | | |
| 1^4^ | observational studies | very serious^i^ | not serious | not serious | not serious | none | In one study, specificity was measured at 98.61% (n=567/579). In other words, 98.61% of children without HIV, tested negative.  ^j^ | | | | ⨁◯◯◯ Very low | IMPORTANT |
| **Accuracy (Sensitivity)** | | | | | | | | | | | | |
| 1^4^ | observational studies | very serious^i^ | not serious | not serious | not serious | none | In one study, sensitivity was measured at 100% (n=4/4). In other words, 100% of children with HIV, tested positive.  ^j^ | | | | ⨁◯◯◯ Very low | CRITICAL |
| **Accuracy (TP, FP, TN, FN, and PPV)** | | | | | | | | | | | | |
| 3^2,3,4^ | observational studies | very serious^k^ | serious^l^ | not serious | not serious | none | Three studies presented accuracy results. A total of 47 True Positives and 81 False Positives were found in the three studies. Only one study conducted confirmatory testing for negative HIV self-tests, and found 567 True Negatives and 0 False Negatives. The pooled positive predictive value across the three studies was 36.72% (n=47/128); ie, 36.72% of children who screened positive were HIV positive.  ^j,m^ | | | | ⨁◯◯◯ Very low | CRITICAL |
| **Accuracy (NPV)** | | | | | | | | | | | | |
| 1^4^ | observational studies | very serious^i^ | not serious | not serious | not serious | none | In one study, the negative predictive value was measured at 100% (n=567/567). In other words, 100% of children screened negative by caregiver-assisted HIV self test, did not have HIV.  ^j^ | | | | ⨁◯◯◯ Very low | IMPORTANT |
| **Feasibility (1): Caregiver correctly performed test** | | | | | | | | | | | | |
| 1^4^ | observational studies | extremely serious^n^ | not serious | not serious | not serious | none | One cohort study where, among those caregivers directly observed, 80.79% (n=635/786) correctly administered the HIV self-test to a child. Among caregivers who had received a demonstration, 92.4% (n=145/157) correctly performed the test. Among caregivers who had not received a demonstration, 77.9% (n=490/629) correctly administered the test. | | | | ⨁◯◯◯ Very low | CRITICAL |
| **Feasibility (2): Caregiver reported test was easy** | | | | | | | | | | | | |
| 2^2,3^ | observational studies | extremely serious^g^ | not serious | not serious | not serious | none | Three cohort studies where, among caregivers interviewed, 92.89% (n=3110/3348) reported it was easy to conduct caregiver-assisted HIV testing with HIVST kit. Among caregivers who had received a demonstration, 96.95% (n=2636/2719) reported HIVST kit was easy to use. Among caregivers who had not received a demonstration, 75.30% (n=474/629) reported HIVST kit was easy to use. | | | | ⨁◯◯◯ Very low | CRITICAL |
| **Positivity (Caregiver-assisted HIVST vs. Provider-administered HCT, among selected testing regimen)** | | | | | | | | | | | | |
| 1^1^ | observational studies | extremely serious^o^ | not serious | not serious | serious^p^ | none | 1/300 (0.3%) | 38/5026 (0.8%) | **RR 0.44** (0.06 to 3.20) | **4 fewer per 1,000** (from 7 fewer to 17 more) | ⨁◯◯◯ Very low | IMPORTANT |
| **Positivity (Caregiver-assisted testing with HIVST vs. Facility-based HTS, among selected testing regimen)** | | | | | | | | | | | | |
| 1^1^ | observational studies | extremely serious^o^ | not serious | not serious | serious^p^ | none | 1/300 (0.3%) | 26/3539 (0.7%) | **RR 0.45** (0.06 to 3.33) | **4 fewer per 1,000** (from 7 fewer to 17 more) | ⨁◯◯◯ Very low | IMPORTANT |
| **Positivity (Caregiver-assisted testing with HIVST vs. Home-based HTS, among selected testing regimen)** | | | | | | | | | | | | |
| 1^1^ | observational studies | extremely serious^o^ | not serious | not serious | serious^p^ | none | 1/300 (0.3%) | 12/1487 (0.8%) | **RR 0.41** (0.05 to 3.16) | **5 fewer per 1,000** (from 8 fewer to 17 more) | ⨁◯◯◯ Very low | IMPORTANT |
| **Positivity (confirmed HIV positive, among enrolled)** | | | | | | | | | | | | |
| 2^2,3^ | observational studies | extremely serious^q^ | not serious | not serious | not serious | none | In two single-arm studies, the pooled HIV prevalence rate was 0.57% (n=43/7608) | | | | ⨁◯◯◯ Very low | IMPORTANT |
| **Linkage to Confirmatory Testing (among enrolled)** | | | | | | | | | | | | |
| 2^2,3^ | observational studies | very serious^r^ | not serious | not serious | not serious | none | In two single-arm studies, among all children enrolled, 1.52% (n=116/7608) were linked to confirmatory testing. | | | | ⨁⨁◯◯ Low^s^ | CRITICAL |
| **Linkage to Confirmatory Testing (among tested positive)** | | | | | | | | | | | | |
| 2^2,3^ | observational studies | extremely serious^t^ | not serious | not serious | not serious | none | In two single-arm studies, among all children screened positive, 97.48% (n=116/119) were linked to confirmatory testing. | | | | ⨁◯◯◯ Very low | CRITICAL |
| **Linkage to Care (among enrolled)** | | | | | | | | | | | | |
| 2^2,3^ | observational studies | extremely serious^u^ | not serious | not serious | not serious | none | In two single-arm studies, among all children enrolled, 0.57% (n=43/7593) were linked to care. | | | | ⨁◯◯◯ Very low | CRITICAL |
| **Linkage to Care (among confirmed positive)** | | | | | | | | | | | | |
| 2^2,3^ | observational studies | extremely serious^v^ | not serious | not serious | not serious | none | In two single-arm studies, among all children confirmed positive, 100% (n=43/43) were linked to care. | | | | ⨁◯◯◯ Very low | CRITICAL |
| **Social Harm** | | | | | | | | | | | | |
| 3^1,3,5^ | observational studies | extremely serious^w^ | not serious | not serious | not serious | none | Across all three studies looking at caregiver-assisted HIV testing with an HIVST kit, no forms of physical harm against a child was reported. In two studies, no forms of physical harm to caregivers as a result of the intervention were reported. In one study, 0.4% (n=9/2250) caregivers reported adverse events affecting children, including 6 who reported pain the gum, 3 reported bleeding of the gum, and 1 reporting itching. | | | | ⨁◯◯◯ Very low | CRITICAL |

**CI:** confidence interval; **RR:** risk ratio

***Explanations***

a. Downgraded 3 levels due to critical risk of bias for 1) deviations from intended interventions (significant cross-overs between arms), 2) attrition (over 20%), and serious risk of bias for 1) confounding factors (gender imbalance), 2) selection of participants into study (facility-based), and 3) self-reported result. Note analysis here follows ITT (denominator: # of children enrolled).

b. Unable to pool results into a meta-analysis. In this study, participants are provided 3 testing options: 1) caregiver-assisted HIVST, 2) facility-based testing, 3) home-based testing. In a two-by-two table comparing intervention arm 1) to the comparators 2) and 3), the denominator would have to be divided. In doing so, the numerators in the comparative arms would be higher than the denominators.

c. Downgraded 3 levels due to critical risk of bias for 1) deviations from intended interventions (significant cross-overs between arms), 2) attrition (over 20%), and serious risk of bias for 1) confounding factors (gender imbalance), 2) selection of participants into study (facility-based), and 3) self-reported result. Note denominator here is not children enrolled, but children per selected testing regimen.

d. Downgraded once. Does not directly answer uptake as analysis is not conducted among total children enrolled, but among children in selected testing regimen. Thus uptake here is measured among participants who already selected and opted for a testing option.

e. Children switched from originally selected testing regimen (ie, selected intervention arm) were excluded

f. Downgraded once as confidence interval crosses line of no effect

g. Downgraded 3 levels due to critical risk of bias due to: 1) bias in selection of participants (facility-based AND self-selected participants), and serious risk of bias due to: 1) confounding (gender imbalance), 2) attrition (7% n=59/867), 4) social desirability bias. Further downgraded 1 level, because this analysis does not follow ITT, but caregivers who were interviewed (LTFU in Tumwesigye, and sample of participants interviewed in Stecker).

h. Downgraded once. Study design does not directly answer acceptability as prior to study enrollment, caregivers were offered 3 testing options, including caregiver-assisted HIV self-testing. Participants wo selected/opted for that test option were enrolled in study. Thus acceptability may appear higher in this group and does not include caregivers who did not accept the intervention.

i. Risk of bias measured by QUADAS-2. Downgraded two levels due to High RoB for 1) patient selection (self-selected, not randomized) 2) Reference standard (no blinding of HCW, moreover 1 study reference test is subjective interpretation), 2) flow and timing: high loss of follow-up.

j. Note that the accuracy results reported for Chikwari, 2019 are different from published results. Measures used here in this analysis were reviewed and validated by study authors.

k. Risk of bias measured by QUADAS-2. Downgraded twice due to High RoB for 1) Patient selection (self-selectd, not randomized), 2) Reference standard (no blinding of HCW doing reference test, moreover 1 study reference test is subjective interpretation), 2) flow and timing: high loss of follow-up. Furthermore, not all index tests were followed by confirmatory testing in 2 of the 3 trials.

l. The PPV measured in Chikwari and Tumwesigye was 33.33% (n=4/12) and 32.65% (n=32/98), respectively. PPV measured in Stecker was 61.11% (n=11/18).

m. Tumwesigye and Stecker et al did not conduct confirmatory testing for children screened negative, so true and false negatives were not tested.

n. Downgraded 2 levels due to serious risk of bias due to: 1) confounding (gender imbalance), 2) bias in selection of participants (facility-based), 3) attrition (7% n=59/867). Note, further downgrade 1 level because this analysis does not follow ITT (867), but caregivers who performed test themselves (786) - thus missing analysis on those caregivers directly observed who did not perform test themselves (22).

o. Downgraded 3 levels due to critical risk of bias for 1) deviations from intended interventions (significant cross-overs between arms), 2) attrition (over 20%), and serious risk of bias for 1) confounding factors (gender imbalance), 2) selection of participants into study (facility-based), and 3) self-reported result. Note: Denominator is not ITT, but number of children in selected testing regimens.

p. Downgraded once due to large confidence interval and confidence interval crossing line of no effect

q. Downgraded 3 levels due to critical risk of bias for 1) selection of participants into study (facility-based AND self-selected participants), and serious risk of bias for 1) confounding factors (gender imbalance), 2) positivity results self-reported. Note denominator not ITT, with children missed in analysis (n=41).

r. Downgraded 2 levels due to critical risk of bias for 1) selection of participants into study (facility-based AND self-selected participants), and serious risk of bias for 1) confounding factors (gender imbalance), 2) positivity results self-reported. Follows ITT analysis.

s. Note that certainty of evidence here is low, not very low, because RoB was downgraded 2 levels not 3. This outcome was not self-reported but measured by clinical data, and follows an ITT analysis.

t. Downgraded 2 levels due to critical risk of bias for 1) selection of participants into study (facility-based AND self-selected participants), and serious risk of bias for 1) confounding factors (gender imbalance), 2) positivity results self-reported. Further downgraded 1 level because denominator here not ITT, but number of children tested/screened positive

u. Downgraded 2 levels due to critical risk of bias for 1) selection of participants into study (facility-based AND self-selected participants), and serious risk of bias for 1) confounding factors (gender imbalance), 2) positivity results self-reported, and 3) timeframe for outcome measurement (1 month and 2 weeks) might not reflect linkage to care that occurred after the reporting period. Further downgraded 1 level because denominator not ITT, and we recognize a number of children missed in analysis.

v. Downgraded 2 levels due to critical risk of bias for 1) selection of participants into study (facility-based AND self-selected participants), and serious risk of bias for 1) confounding factors (gender imbalance), 2) positivity results self-reported, and 3) timeframe for outcome measurement (1 month and 2 weeks) might not reflect LtC that occurred after the reporting period. Further downgraded 1 level because denominator not ITT but children confirmed positive.

w. Critical and serious risk of bias for 1) confounding factors for all 3 studies (gender imbalance), 2) selection of participants for 2 studies where caregivers were pre-screened for IPV prior to intervention (226 or 5.3% of caregivers were screened-out); 3) deviations for 1 study (significant cross-arm switches), 4) Missing data for 1 study with 27% attrition rate; 5) Measurement of outcomes: critical for all 3 as contingent self-reporting; Note: Denominator here not ITT, but number of persons interviewed.

***References***

1. Dziva Chikwari C, Simms V, Kranzer K, Dringus S, Chikodzore R, Sibanda E, Webb K, Engelsmann B, Redzo N, Bandason T, Mujuru H, Apollo T, Ncube G, Hatzold K, Weiss HA, Ferrand RA. Comparison of index-linked HIV testing for children and adolescents in health facility and community settings in Zimbabwe: findings from the interventional B-GAP study. Lancet HIV. 2021;8(3):e138-e48.

2. Steker C. Adding to the HIV Testing Services Toolkit! Caregiver-Assisted Oral HIV Screening of Children 18 Months – 14 Years in Uganda and Zambia. International Workshop on HIV & Pediatrics; 28 July 2022;Montreal, Canada.

3. Tumwesigye N, Stecker C, Biribawa C, Mukose A, Namanda C, Oliver D, Mwangi C, Mabirizi D, Awor A, Nazziwa E, Okello F, Alwano M, Hast M, Gross J, Parris K, Taasi G, Parekh B. Performance evaluation between home-based caregiver-assisted oral HIV screening of children and facility-based confirmatory testing using the national algorithm in Uganda. 24th International AIDS Conference; 29 July - 2 August 2022;Montreal, Canada.

4. Dziva Chikwari C, Simms V, Kranzer K, Dringus S, Chikodzore R, Sibanda E, Webb K, Redzo N, Mujuru H, Apollo T, Ncube G, Hatzold K, Bernays S, Weiss HA, Ferrand RA. Feasibility and Accuracy of HIV Testing of Children by Caregivers Using Oral Mucosal Transudate HIV Tests. J Acquir Immune Defic Syndr. 2021;87(2):781-8.

5. Gross J, Parris K, Okello F, Taasi G, Magongo E, Matovu J, Tumwesigye N, Kagaayi J, Mukose A, Biribawa C, Kagongwe S, Nabuduwa A, Namanda C, Kaakyo M, Nsenga M, Komukama C, Nazziwa E, Adler M, Apolot M, Alwano M, Zyambo Z, Munthali G, Mwiya M, Mutembo S, Moyo N, Matoba J, Chilyabanyama O, Ndubani P, Itoh E, Boyd A, Musokotwane K, Chungu C, Hast M, Rivadeneira M, Oliver D, Lyon T, Fenn T, Stecker C. Minimizing the risk of social harm related to caregiver-assisted oral HIV self-testing (HIVST) to screen children of people living with HIV. International Workshop on HIV & Pediatrics 28 July 2022;Montreal, Canada
