# Supplementary material for: Caregiver-assisted testing with HIV self-test kits for children 18 months and older: A GRADE systematic review
Source: PLOS Glob Public Health. 2024 Aug 14;4(8):e0003588. doi: 10.1371/journal.pgph.0003588 (PMC11324119; doi:10.1371/journal.pgph.0003588)
Supplement: S3 Table — This appendix contains the subgroup analyses for each outcome assessed by gender and children age groups. (DOCX) [file pgph.0003588.s003.docx]

### **S4 Table. Within-Study Subgroup Analyses**

| *Subgroup analysis of HIV testing uptake: female vs. male children* | | | | | | |
| --- | --- | --- | --- | --- | --- | --- |
| **#** | **Study Year** | **Country** | **ID** | **Arms** | **Intervention** | **Comparators** |
| 1a | Chikwari, 2021 | Zimbabwe | B-GAP | 3 | **CG-HIVST**  F: 111/3115 (3.56%)  M: 89/2940 (3.03%)  (7 unknown gender) | **1) FB-HCT**  F: 1050/3115 (33.71%)  M: 863/2940 (29.35%)  (3 unknown gender) |
| 1b |  |  |  |  |  | **2) HB-HCT**  F: 750/3115 (24.08%)  M: 768/2940 (26.12%)  (4 unknown gender) |
| *Subgroup analysis of HIV testing uptake: children age groups* | | | | | | |
| **#** | **Study Year** | **Country** | **ID** | **Arms** | **Intervention** | **Comparator** |
| 1a | Chikwari, 2021 | Zimbabwe | B-GAP | 3 | **CG-HIVST**  2-4 yrs: 44/1284 (3.43%)  5-9 yrs: 62/2103 (2.95%)  10-14 yrs: 71/1738 (4.09%)  15-18 yrs: 23/937 (2.45%) | **1) FB-HCT**  2-4 yrs: 495/1284 (38.55%)  5-9 yrs: 679/2103 (33.14%)  10-14 yrs:506/1738 (29.11%)  15-18 yrs: 236/937 (25.19%) |
| 1b |  |  |  |  |  | **2) HB-HCT**  2-4 yrs: 306/1284 (23.83%)  5-9 yrs: 529/2103 (25.15%)  10-14 yrs: 452/1738 (26.0%)  15-18 yrs: 235/937 (25.08%) |
| *Subgroup analysis of HIV positivity (tested positive, among children tested) : female vs. male children* | | | | | | |
| **#** | **Study Year** | **Country** | **ID** | **Arms** | **Intervention** | **Comparator** |
| 1a | Chikwari, 2021 | Zimbabwe | B-GAP | 3 | **CG-HIVST**  F: 1/111 (0.90%)  M: 0/89 (0%) | **1) FB-HCT**  F: 19/1050 (1.81%)  M: 7/863 (0.81%)  (0 among unknown gender) |
| 1b |  |  |  |  |  | **2) HB-HCT**  F: 8/750 (1.07%)  M: 4/768 (0.52%)  (0 among unknown gender) |
| *Subgroup analysis of HIV positivity (tested positive, among children tested): children age groups* | | | | | | |
| **#** | **Study Year** | **Country** | **ID** | **Arms** | **Intervention** | **Comparator** |
| 1a | Chikwari, 2021 | Zimbabwe | B-GAP | 3 | **CG-HIVST**  2-4 yrs: 0/44 (0%)  5-9 yrs: 0/62 (0%)  10-14 yrs: 1/71 (1.41%)  15-18 yrs: 0/23 (0%) | **1) FB-HCT**  2-4 yrs: 2/495 (0.4%)  5-9 yrs: 6/680 (0.88%)  10-14 yrs: 8/507 (1.58%)  15-18 yrs: 10/236 (4.24%) |
| 1b |  |  |  |  |  | **2) HB-HCT**  2-4 yrs: 0/306 (0%)  5-9 yrs: 5/529 (0.95%)  10-14 yrs: 6/452 (1.33%)  15-18 yrs: 1/235 (0.43%) |
| *Subgroup analysis of HIV positivity (confirmed positive, among children enrolled) : female vs. male children* | | | | | | |
| **#** | **Study Year** | **Country** | **ID** | **Arms** | **Intervention** | **Comparator** |
| 1 | Tumwesigye, 2022 | Uganda | FASTER | 1 | **CG-HIVST**  *F: 18/2440 (0.74%)*  *M: 14/2425 (0.58%)** | - |
| 2 | Stecker, 2022 | Zambia |  | 1 | **CG-HIVST**  F: 6/1343 (0.45%)  M: 5/1400 (0.36%) | - |
| *Subgroup analysis of HIV positivity (confirmed positive, among children enrolled): children age groups* | | | | | | |
| **#** | **Study Year** | **Country** | **ID** | **Arms** | **Intervention** | **Comparator** |
| 1 | Tumwesigye, 2022 | Uganda | FASTER | 1 | **CG-HIVST**  18-23 mo: 2/106 (2.24%)  2-4 yrs: 15/1144 (1.31%)  5-9 yrs: 27/1982 (1.36%)  10-14 yrs: 22/1633 (1.34%) | - |
| 2 | Stecker, 2022 | Zambia |  | 1 | **CG-HIVST**  18 -23 mo: 1/126 (0.79%)  2-4 yrs: 2/628 (0.32%)  5-9 yrs: 4/1087 (0.37%)  10-14 yrs: 4/886 (0.45%) | - |
| *CG-HIVST: Caregiver-assisted HIV testing with HIV self-test kit; FB-HCT: facility-based HIV Counseling and Test; HB-HCT: home-based HIV Counseling and Test* | | | | | | |
