# Supplementary material for: Caregiver-assisted testing with HIV self-test kits for children 18 months and older: A GRADE systematic review
Source: PLOS Glob Public Health. 2024 Aug 14;4(8):e0003588. doi: 10.1371/journal.pgph.0003588 (PMC11324119; doi:10.1371/journal.pgph.0003588)
Supplement: S4 Table — This appendix contains the summary of the reported unit costs for caregiver-assisted HIV self-testing, expressed in 2021 USD. Comparative unit costs for alternative index-testing modalities were included when reported. (DOCX) [file pgph.0003588.s004.docx]

**S5 Table. Summary of costs, in 2021 USD**

| **Study** | **Country** | **Type** | **Cost per child tested** | | | | **Cost per child diagnosed HIV positive** | | | |
| --- | --- | --- | --- | --- | --- | --- | --- | --- | --- | --- |
|  | | | SOC | **CG-HIVST** | FB-HTS | HB-HTS | SOC | **CG-HIVST** | FB-HTS | HB-HTS |
| Vasantharoopan et al., 2021 *(16)* | Zimbabwe | Inc; Fin | 14.58-17.67 | **43.48-154.23** | 12.22-76.82 | 16.54-21.38 | 345.43-488.89 | **2467.68** | 871.47-3687.70 | 2019.83-3819.66 |
| Kagaayi et al., 2022 *(19)* | Uganda | Full; Fin |  | **5.80** |  |  |  | **869.00** |  |  |
|  | Zambia |  |  | **5.12** |  |  |  | **1,191.00** |  |  |
| *Prices inflated in local currency and then converted to 2021 USD as recommended by Kumaranayake, 2000 [(26)](https://sciwheel.com/work/citation?ids=14233525&pre=&suf=&sa=0&dbf=0). Note that prices in Zimbabwe may appear high due to country’s hyper-inflation.  *Inc: Incremental; Fin: Financial; CG: Caregiver; HIVST: HIV self-testing; FB: Facility-based; HB: Home-based; HTS: HIV testing services; SOC: standard of care; USD: United States Dollars* | | | | | | | | | | |
