# Supplementary material for: Caregiver-assisted testing with HIV self-test kits for children 18 months and older: A GRADE systematic review
Source: PLOS Glob Public Health. 2024 Aug 14;4(8):e0003588. doi: 10.1371/journal.pgph.0003588 (PMC11324119; doi:10.1371/journal.pgph.0003588)
Supplement: S1 Text — This appendix contains the risk of bias assessment for each included study using the ROBINS-1 and QUADAS-2 assessment tools. It includes summary results as well as a detailed justification of the assessment for each study and each domain. (DOCX) [file pgph.0003588.s005.docx]

### **S2. Risk of Bias Assessment**

**S2 Figure A. Risk of bias graph and summary: review of authors' judgements about each domain**

| *ROBINS-1 Assessment for observational studies*   \|  \| Confounding \| Selection of participants \| Classification \| Deviations \| Missing data \| Outcomes measurement \| Selective reporting \| \| --- \| --- \| --- \| --- \| --- \| --- \| --- \| --- \| \| Chikwari, 2021 \| - \| - \| + \| -- \| -- \| - \| - \| \| Tumwesigye, 2022 \| - \| -- \| + \| + \| + \| - \|  \| \| Stecker, 2022 \| - \| -- \| + \| + \| + \| - \|  \|  \| + \| Low risk \|  \| - \| Moderate risk \|  \| - \| Serious risk \|  \| -- \| Critical risk \|  \|  \| No Information \| \| --- \| --- \| --- \| --- \| --- \| --- \| --- \| --- \| --- \| --- \| --- \| --- \| --- \| --- \|     *QUADAS-2 Assessment for Diagnostic Accuracy*  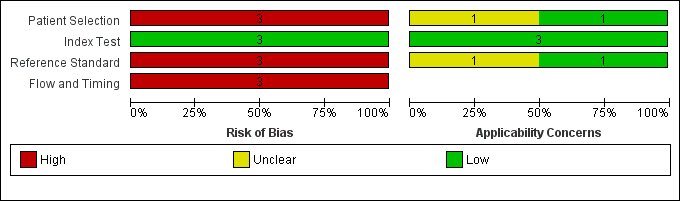  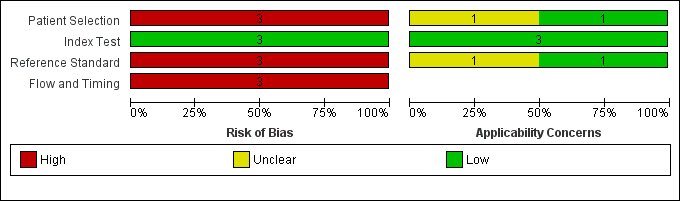  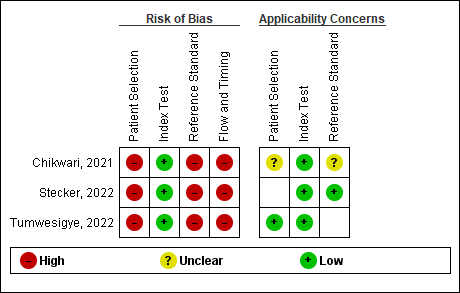  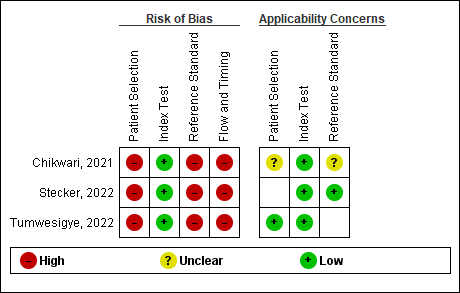 |
| --- | --- | --- | --- | --- | --- | --- | --- | --- | --- | --- | --- | --- | --- | --- | --- | --- | --- | --- | --- | --- | --- | --- | --- | --- | --- | --- | --- | --- | --- | --- | --- | --- | --- | --- | --- | --- | --- | --- | --- | --- | --- | --- | --- | --- | --- | --- |

**S2 Table A. ROBINS-I Risk of bias table by study**

| **ROBINS-I Risk of Bias Assessment for observational studies** | |
| --- | --- |
| Author/Year | **Chikwari et al, 2021 *(5)*** |
| Bias due to confounding | **Serious**  Significant gender imbalance in participants’ demographic profile (21.2% male, 78.8% female). Multivariate analysis further confirms women are more likely to uptake – thus testing uptake and ensuing outcomes may appear greater among this study population. |
| Bias in selection of participants | **Serious**  Caregivers recruited from HIV/ART health facilities, thus precluding undiagnosed PLHIV or diagnosed PLHIV not accessing or adhering to HIV care at health facilities. Effect: precludes families potentially at greater risk of HIV transmission to child. |
| Bias in classification | **Low**  Low risk of incomplete recording of interventions and outcomes |
| Bias due to deviations from intended interventions | **Critical**  Participants and study staff not blind to intervention arms. 71 switched to facility testing, and 3473 switched from facility to community testing arms. |
| Bias due to missing data | **Critical**  Significant attrition of 21%: 1101 children enrolled were not found during tracing, 19 children tested with unknown test outcome. Facility arm attrition: 35% (n=1221/3539). Community-based arms attrition: 26% (n=467/1787). No further information provided on attrition between home-based and caregiver arms. |
| Bias in measurement of outcomes | **Serious**  Measure of testing uptake, positivity, acceptability, and feasibility, and social harm contingent on caregivers self-reporting. |
| Bias in selection of the reported result | **Low**  All reported results correspond to intended outcomes stated in protocol. |
|  |  |
| Author/Year | **Tumwesigye et al, 2022 *(17)*** |
| Bias due to confounding | **Serious**  Significant gender imbalance in participants’ demographic profile (26.8% male, 73.2% female for caregivers). Multivariate analysis further confirms women are more likely to uptake – thus testing uptake and ensuing outcomes may appear greater among this study population. |
| Bias in selection of participants | **Critical**  Caregivers recruited from HIV/ART health facilities, thus precluding undiagnosed PLHIV or diagnosed PLHIV not accessing or adhering to HIV care at health facilities. Effect: precludes families potentially at greater risk of HIV transmission to child. Moreover, participants enrolled are participants who opted into the intervention. Participants who did not opt for intervention were directed to other services and excluded from study. |
| Bias in classification | **Low**  Low risk of incomplete recording of interventions and outcomes |
| Bias due to deviations from intended interventions | **Low**  Single-arm trial |
| Bias due to missing data | **Low**  Small attrition of 2.9% (67/2318). |
| Bias in measurement of outcomes | **Serious**  Measure of testing uptake, positivity, acceptability, and feasibility, and social harm contingent on caregivers self-reporting. |
| Bias in selection of the reported result | **No Information**  No protocol available to make judgement |
|  |  |
| Author/Year | **Stecker et al, 2022 *(8)*** |
| Bias due to confounding | **Serious**  Significant gender imbalance in participants’ demographic profile (12.6% male, 87.5% female). Multivariate analysis further confirms women are more likely to uptake – thus testing uptake and ensuing outcomes may appear greater among this study population. |
| Bias in selection of participants | **Critical**  Caregivers recruited from HIV/ART health facilities, thus precluding undiagnosed PLHIV or diagnosed PLHIV not accessing or adhering to HIV care at health facilities. Effect: precludes families potentially at greater risk of HIV transmission to child. Moreover, participants enrolled are participants who opted into the intervention. Participants who did not opt for intervention were directed to other services and excluded from study. |
| Bias in classification | **Low**  Low risk of incomplete recording of interventions and outcomes |
| Bias due to deviations from intended interventions | **Low**  Single-arm trial |
| Bias due to missing data | **Low**  Small attrition of 3.7% (59/1611). |
| Bias in measurement of outcomes | **Serious**  Measure of testing uptake, positivity, acceptability, and feasibility, and social harm contingent on caregivers self-reporting. |
| Bias in selection of the reported result | **No Information**  No protocol available to make judgement |

**S2 Table B. QUADAS-2 Risk of bias table by study**

| **QUADAS-2 Risk of Bias Assessment for Diagnostic Accuracy** | |
| --- | --- |
| Author/Year | **Chikwari et al, 2021 (b) *(6)*** |
| Patient selection | **High risk**  Participants self-selected, not randomized. |
| Index test | **Low risk**  Index test results were interpreted without knowledge of reference standard results. |
| Reference standard | **High risk**  Reference standard results interpreted by a health-care worker with knowledge of results of index test. Reference standard is research assistant’s interpretation of an HIV self-test result, not the result of a confirmatory rapid diagnostic test. Though health care workers’ interpretations were noted as having 100% specificity and sensitivity, the comparator is subjective and not the same reference standard used in the FASTER studies. |
| Flow and timing | **High risk**  Interpretation data was available for 75% (n= 587/786) of tests, resulting in high loss-to-follow-up of 25%. |
|  |  |
| Author/Year | **Tumwesigye et al, 2022 *(11, 17)*** |
| Patient selection | **High risk**  Participants self-selected, not randomized. |
| Index test | **Low risk**  Index test results were interpreted without knowledge of reference standard results. |
| Reference standard | **High risk**  Reference standard results interpreted by a health-care worker with knowledge of results of index test. Reference standard followed national algorithm and likely correctly classified results. |
| Flow and timing | **High risk**  Only patients tested positive with and HIVST kit received confirmatory testing by reference standard. |
|  |  |
| Author/Year | **Stecker et al, 2022 *(8)*** |
| Patient selection | **High risk**  Participants self-selected, not randomized. |
| Index test | **Low risk**  Index test results were interpreted without knowledge of reference standard results. |
| Reference standard | **High risk**  Reference standard results interpreted by a health-care worker with knowledge of results of index test. Reference standard followed national algorithm and likely correctly classified results. |
| Flow and timing | **High risk**  Only patients tested positive with and HIVST kit received confirmatory testing by reference standard. |
